# Distal Third Minimally Displaced Stress Fracture of Fibula in a Military Recruit: A Case Report and Review of Literature

**DOI:** 10.1155/cro/3931817

**Published:** 2026-06-22

**Authors:** Arjun Poudel, Bimal Rai, Deekshya Devkota, Vinayak Dhungana

**Affiliations:** ^1^ Department of Orthopedic Surgery, Shree Birendra Hospital, Kathmandu, Nepal; ^2^ Department of Pediatric Surgery, Kanti Children′s Hospital, Kathmandu, Nepal

**Keywords:** case report, displaced, distal fibula, military, ORIF, stress fracture

## Abstract

**Background:**

Stress fractures frequently occur in athletes and military personnel due to their rigorous training and are typically treated conservatively with immobilization and rehabilitation. However, a displaced distal third stress fracture of the fibula that requires surgical intervention is a rare occurrence.

**Case Presentation:**

A 20‐year‐old male military recruit under training for 1 month presented with a week‐long history of pain and swelling in his right ankle and difficulty in walking. A plain radiograph revealed a transverse fracture of the fibula with a minimal displacement, consistent with a Kaeding–Miller type III stress fracture. Although considered low‐risk, the fracture was treated surgically with open reduction and internal fixation using a one‐third tubular plate due to the rigorous demands of military training and the extended recovery time associated with conservative management. Postoperatively, nonweight bearing with structured rehabilitation was done, and the fracture healed early without complications.

**Discussion:**

A minimally displaced stress fracture of the distal fibula managed operatively is a rare entity with very limited literature published which poses diagnostic and therapeutic challenges. This case demonstrates that minimally displaced fibular stress fractures can be diagnosed using plain radiographs and may necessitate operative reduction and fixation, especially in high‐demand athletes and military personnel.

**Highlights**

1. Distal fibular stress fractures are rare, accounting for a small proportion of lower extremity stress injuries and often pose diagnostic and management challenges.
2. We report a minimally displaced (Kaeding–Miller type III) distal fibular stress fracture in a young military recruit following repetitive high‐impact training.
3. Despite being a low‐risk fracture, surgical management with open reduction and internal fixation (ORIF) was chosen due to high functional demands.
4. Operative management combined with structured rehabilitation resulted in rapid recovery and excellent functional outcome.
5. This case highlights that operative management may be considered in selected low‐risk stress fractures to facilitate early return to activity in high‐demand individuals.

## 1. Introduction

A stress fracture is a well‐known condition commonly found in healthy athletes and military personnel, which can be attributed to the training process that encompasses physical stresses and exertion [1]. These fractures, which can be either partial or complete, occur due to the imbalance between osteoclastic bone resorption and osteoblastic bone formation during remodeling. When osteoclastic activity predominates in response to mechanical loading, there is a temporary phase of reduced bone strength, elevating the fracture risk until osteoblastic repair re‐establishes bone mineral density and structural integrity [2]. Kaeding and Miller have proposed a classification system for stress fractures based on the clinical features and radiographic findings [3].

A distal fibular stress fracture is a relatively uncommon site injury that can occur in military personnel undergoing intense physical training [4]. A systematic review conducted by Encinas et al. [5] revealed that fibula stress fractures account for approximately 8.6% of all stress fractures. Literature regarding minimally displaced distal fibular stress fractures is sparse, and limited to case reports and small‐scale studies, thus posing significant challenges in diagnosis and management. Timely diagnosis and management are important to ensure effective recovery from these injuries [3].

We present a case of an adult male military recruit who developed a minimally displaced distal fibula stress fracture after vigorous exercise including running and jumping during military training. This case report presents the patient′s clinical presentation, radiological findings, surgical management, and rehabilitation protocol. We intend to add to the sparse body of literature regarding the diagnosis and surgical management of minimally displaced distal fibular stress fractures to ensure optimal recovery and timely return to activity.

The report adheres to the SCARE 2025 guidelines and presents the patient′s clinical presentation, investigations, management, and outcomes [6].

## 2. Case Presentation

A 20‐year‐old male military recruit under training for 1 month presented with a 1‐week history of pain and swelling in his right ankle and difficulty walking. He reported running long distances every morning as part of his military training, without adequate rest and continued running despite experiencing pain in his ankle. The repetitive high‐impact activity caused chronic stress on the fibula, resulting in a stress fracture. The patient complained of pain in his right ankle while weight bearing.

**Figure 1:**
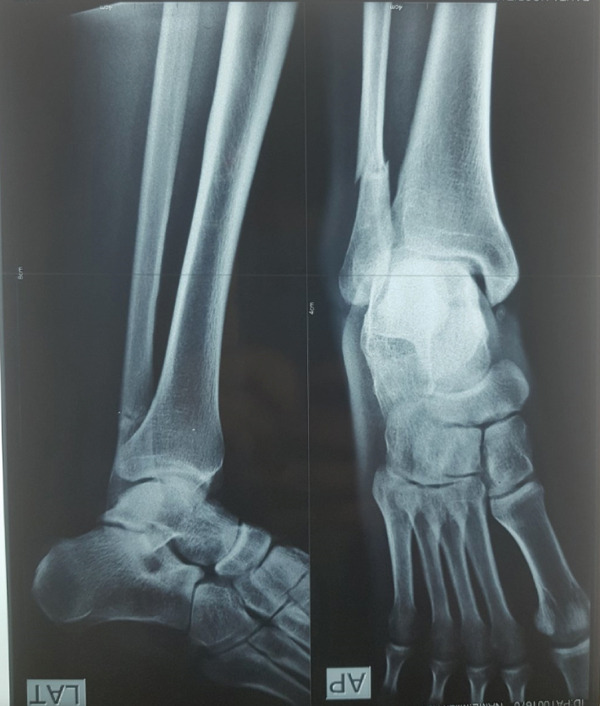
Anteroposterior and lateral plain radiographs showing transverse fracture of the right distal fibula around 5 cm proximal to the lateral malleolus with minimal displacement.

Upon presentation, his vital signs were stable, and physical examination revealed localized swelling and tenderness approximately 5 cm proximal to the lateral malleolus, without any gross visible deformity. The ankle′s range of motion was preserved, and distal neurovascular structures were intact. Anteroposterior and lateral plain radiographs of the ankle revealed a transverse fibular fracture and minimal displacement (less than 2 mm) present 5 cm proximal to the lateral malleolus, consistent with a Kaeding–Miller type III stress fracture (Figure 1).

**Figure 2:**
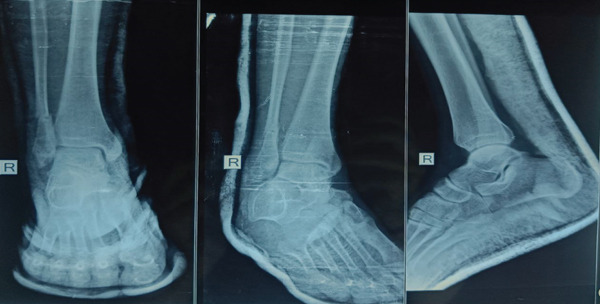
Anteroposterior, mortise, and lateral plain radiographs showing the patient′s right limb immobilized in a below‐knee slab.

The patient′s limb was initially immobilized using a below‐knee posterior slab (Figure 2). Given the demanding nature of military training and the prolonged recovery associated with conservative management, the patient chose surgical intervention despite the fracture being low‐risk.

**Figure 3:**
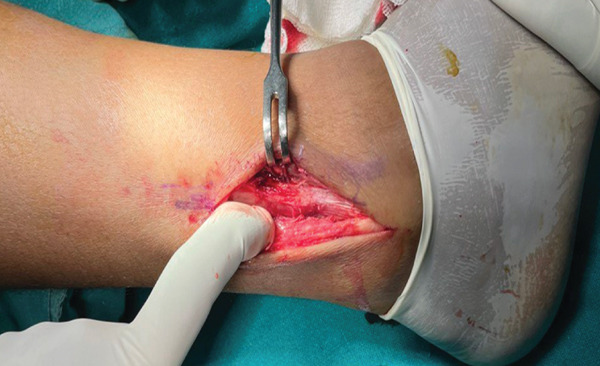
Intraoperative image showing the fracture line.

**Figure 4:**
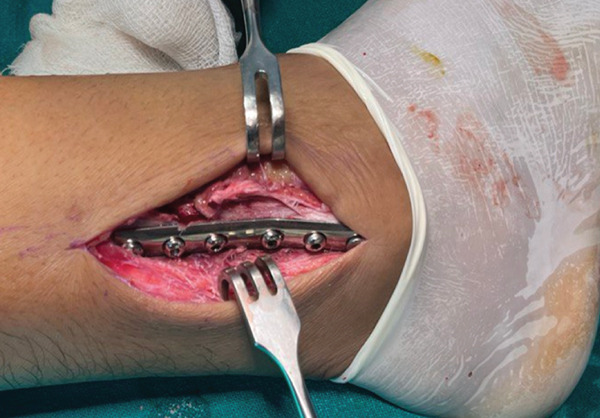
Intraoperative image after in situ fixation with one‐third‐tubular plate.

**Figure 5:**
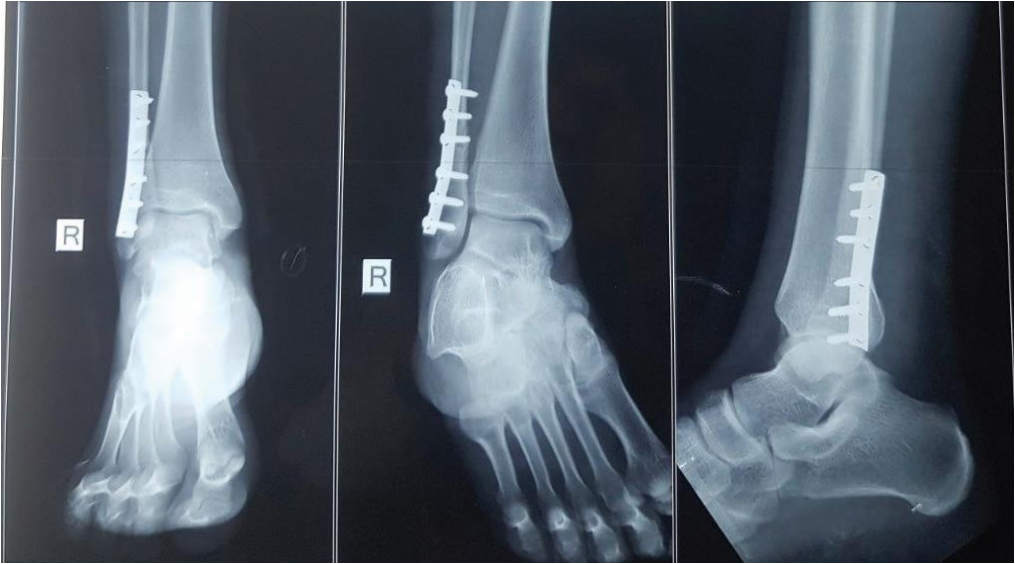
Anteroposterior, mortise, and lateral plain radiographs showing fixation with a one‐third‐tubular plate with six screws.

Preoperative laboratory tests revealed elevated liver enzymes—55.4 U/L for aspartate transaminase (reference range, 13–40 U/L) and 53 U/L for alanine transaminase (reference range, 13–40 U/L) with a negative C‐reactive protein. The surgery was performed under a sciatic nerve block with the patient in the supine position via a direct subcutaneous lateral approach. A 10‐cm incision was made along the posterior border of the distal third of the fibula, and following soft tissue dissection, in situ plate fixation was performed using a titanium six‐hole one‐third‐tubular plate using six titanium screws (Figures 3, 4, and 5). Postoperatively nonweight bearing was done.

The rehabilitation program involved nonweight‐bearing for 2 weeks to promote healing. However, ankle range‐of‐motion exercises including dorsiflexion–plantarflexion and inversion–eversion movements were introduced in the immediate postoperative period to maintain ankle mobility. The patient was refrained from intense military training for 8 weeks until complete fracture healing.

**Figure 6:**
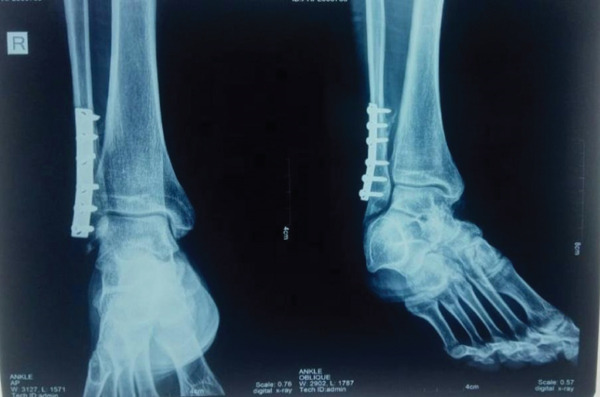
Anteroposterior and mortise plain radiographs of right ankle showing fracture alignment with a semitubular plate in situ post‐4 months of surgery.

Surgical management with structured rehabilitation led to progressive improvement in pain and function. VAS scores decreased from 7/10 at rest (9/10 on loading) preoperatively to 2/10 at 2 weeks postoperatively, and 0/10 by Week 8. The Olerud–Molander Ankle Score improved from 30/100 to 90/100 in 8 weeks, demonstrating excellent functional recovery. The patient commenced jogging at Week 10 and returned to unrestricted running at Week 12. Full military duty, including high‐impact drills, was resumed at 14 weeks postoperatively with no recurrence of symptoms. Clinical and radiological examinations during a 4‐month follow‐up showed satisfactory fracture alignment and callus formation, with no complications (Figure 6).

## 3. Discussion

Stress fractures generally occur due to repetitive, cyclic loading which exceeds the mechanical capacity of the bone. Stress fractures of the femoral neck, patella, medial malleolus, anterior tibial cortex, and so on, are considered high‐risk with the risk of delayed union or nonunion, risk of refracture, and other long‐term complications. Low‐risk stress fractures including femoral shaft, medial tibia, ribs, ulnar shaft, fibula, and so on, have less chance of complications and are managed conservatively [7]. However, in our case, a minimally displaced distal third stress fracture of the fibula, considered low‐risk, is managed operatively.

Approximately 95% of all stress fractures occur in the lower extremities, more commonly involving the tibia, with less than 20% occurring in the fibula [8, 9]. In our case, an isolated minimally displaced stress fracture was located in the distal one‐third of the fibula, which is low risk and an unusual presentation given the limited amount of weight‐bearing stress on the fibula.

Our patient is a new military recruit with a rigorous training involving high‐impact activities in his daily life and is at increased risk for stress fracture. Although abundant literature exists on stress fractures involving the tibia, neck of the femur, and metatarsal bones [10], research regarding stress fractures of the fibula remains limited. Only a few case reports have been documented.

A structured literature search was performed in PubMed, Embase, and Google Scholar using the terms “distal fibula stress fracture,” “fibular stress fracture military,” “fibular stress fracture ORIF,” and “fibular stress fracture management”. All English language case reports and small series reporting distal fibular stress fractures with documented management and outcomes were included (Table 1).

**Table 1:** Summary of reported distal fibula stress fracture cases and management.

Author (year)	Patient	Fracture type	Management	Return to activity	Outcome
Cheng et al. (2015) [11]	44F, flatfoot	Nondisplaced distal fibula SF	Conservative (cast, NWB)	~12 weeks	Healed, no complications
Hoglund et al. (2015) [12]	52F, recreational runner	Nondisplaced distal fibula SF	Conservative (boot, PWB)	~10–12 weeks	Full recovery, returned to running
Tavakkolizadeh et al. (2005) [13]	38F, osteoporosis, bilateral	Nondisplaced bilateral distal fibula SF	Conservative + Vit D/Ca supplementation	~12 weeks	Healed, no complications
Meador and Vincent (2019) [14]	21M, collegiate basketball	Displaced distal fibula SF	Surgical (IM screw)	6 weeks to sport	Excellent functional outcome
Present case (2024)	20M, military recruit	Minimally displaced (KM III) distal fibula SF	Surgical (1/3 tubular plate, ORIF)	12 weeks to running; 14 weeks full duty	OMAS 90/100; VAS 0/10 at 8 weeks

Abbreviations: IM, intramedullary; KM, Kaeding–Miller; NWB, nonweight‐bearing; OMAS, Olerud–Molander Ankle Score; PWB, protected weight‐bearing; SF, stress fracture; VAS, visual analog scale.

Cheng et al. [11] reported a case of a 44‐year‐old female with flatfoot presenting with a distal fibular stress fracture that was managed conservatively. Similarly, Hoglund et al. [12] described a 52‐year‐old female recreational runner with a distal fibular stress fracture, also treated conservatively. Tavakkolizadeh et al. [13] documented a case of a 38‐year‐old osteoporotic female who sustained bilateral distal fibular fractures where the patient was managed conservatively with immobilization and supplementation of vitamin D and calcium.

Meador and Vincent [14] described a 21‐year‐old basketball player with a distal fibular stress fracture who underwent early surgical management with intramedullary screw fixation of the left fibula, resulting in faster recovery and improved functional outcomes.

In earlier documented cases, nondisplaced fractures were treated conservatively with nonweight‐bearing regimens, immobilization, and supportive care. However, we present a case involving a minimally displaced stress fracture of the right distal fibula, which was identified using plain radiography. There was a transverse fibular fracture with minimal displacement, indicating a Kaeding–Miller type III. This fracture necessitated surgical intervention and was effectively treated with ORIF with a one‐third tubular plate. Due to the demanding nature of military training, a surgical treatment strategy was adopted, which resulted in early recovery and good functional outcome. This demonstrates that stress fractures should be addressed both operatively and nonoperatively depending on the nature of the fracture and patient characteristics [15].

Although intramedullary screw fixation remains an acceptable alternative for more proximal or middiaphyseal stress fractures, plate fixation of distal fibular fractures provides rigid, stable constructs that safely allow early or immediate full weight‐bearing, with high union rates and low mechanical failure when modern locking plates are used [16].

In a young recruit with lateral ankle pain, multiple differentials were considered and systematically ruled out: ligament sprain (no acute inversion/instability), peroneal tendon pathology (no tendon‐specific signs), sinus tarsi syndrome (no characteristic pain), and osteochondral or subtalar lesions (no deep joint symptoms) [17].

This case highlights the importance of surgical management for stress fractures, even when minimal displacement is present depending upon the patient characteristics. ORIF provided excellent alignment and stability, enabling a quick recovery. This approach emphasizes that although conservative management remains the mainstay for nondisplaced fibular stress fractures, operative intervention should be considered for minimally displaced fractures to ensure optimal functional recovery and minimize complications. Postoperatively, the patient underwent a structured rehabilitation program to facilitate healing and restore function.

The lack of advanced imaging like magnetic resonance imaging (MRI) and metabolic evaluation is a major limitation of this case report. Extending follow‐up to a minimum of 12 months would have strengthened the assessment of implant‐related complications. In our case, the fracture line was visualized on plain radiography; therefore, MRI was not performed. However, in lower grade stress fractures where plain radiographs may be normal, MRI is the imaging modality of choice for confirming the diagnosis in patients with clinical suspicion.

## 4. Conclusion

Although most low‐risk fractures respond well to conservative management, higher grade injuries may require surgical intervention to achieve optimal alignment and facilitate early functional recovery. This case demonstrates that ORIF combined with a structured rehabilitation program can lead to early recovery and excellent functional outcomes in low‐risk stress fractures, especially in high‐demand athletes and military personnel.

## Funding

No funding was received for this manuscript.

## Disclosure

The authors are accountable for all aspects of the work and ensure that questions related to the accuracy or integrity of any part of the work are appropriately investigated and resolved.

## Ethics Statement

All procedures performed in this study were per the ethical standards of the institutional and national research committee(s) and with the Helsinki Declaration (as revised in 2013). Written informed consent was obtained from the patient to publish this case report and accompanying images. All identifying information in the provided images has been removed for patient anonymity. A copy of the written consent is available for review by the editorial office of this journal.

## Conflicts of Interest

The authors declare no conflicts of interest.

## Supporting information


**Supporting Information** Additional supporting information can be found online in the Supporting Information section. File S1: “SCARE‐Checklist‐2025 (2).docx” contains the completed SCARE 2025 checklist for this case report.

## Data Availability

The data that support the findings of this study are available from the corresponding author upon reasonable request.
